# Hypothalamic TRH Mediates Anorectic Effects of Serotonin in Rats

**DOI:** 10.1523/ENEURO.0077-22.2022

**Published:** 2022-05-26

**Authors:** Jorge Chávez, Viridiana Alcántara-Alonso, Cinthia García-Luna, Paulina Soberanes-Chávez, Dimitris Grammatopoulos, Patricia de Gortari

**Affiliations:** 1 Molecular Neurophysiology laboratory, Department of Neuroscience, National Institute of Psychiatry “Ramón de la Fuente Muñiz”, Mexico City 14370, Mexico; 2 Translational Medicine, Warwick Medical School, Coventry CV4 7HL, United Kingdom; 3 Institute of Precision Diagnostics and Translational Medicine, Division of Pathology, University Hospitals Coventry and Warwickshire National Health Service Trust, Coventry CV2 2DX, United Kingdom

**Keywords:** anorexia, PKC, PVN, serotonin, TRH

## Abstract

Among the modulatory functions of thyrotropin-releasing hormone (TRH), an anorectic behavior in rodents is observed when centrally injected. Hypothalamic paraventricular nucleus (PVN) neurons receive serotonergic inputs from dorsal raphe nucleus and express serotonin (5HT) receptors such as 5HT_1A_, 5HT_2A/2C_, 5HT_6_, which are involved in 5HT-induced feeding regulation. Rats subjected to dehydration-induced anorexia (DIA) model show increased PVN TRH mRNA expression, associated with their decreased food intake. We analyzed whether 5HT input is implicated in the enhanced PVN TRH transcription that anorectic rats exhibit, given that 5HT increases TRH expression and release when studied *in vitro*. By using mHypoA-2/30 hypothalamic cell cultures, we found that 5HT stimulated TRH mRNA, pCREB, and pERK1/2 levels. By inhibiting basal PKA or PKC activities or those induced by 5HT, pCREB or pERK1/2 content did not increase suggesting involvement of both kinases in their phosphorylation. 5HT effect on TRH mRNA was not affected by PKA inhibition, but it diminished in the presence of PKCi suggesting involvement of PKC in 5HT-induced TRH increased transcription. This likely involves 5HT_2A/2C_ and the activation of alternative transduction pathways than those studied here. In agreement with the *in vitro* data, we found that injecting 5HT_2A/2C_ antagonists into the PVN of DIA rats reversed the increased TRH expression of anorectic animals, as well as their decreased food intake; also, the agonist reduced food intake of hungry restricted animals along with elevated PVN TRH mRNA levels. Our results support that the anorectic effects of serotonin are mediated by PVN TRH in this model.

## Visual Abstract

**Figure:**
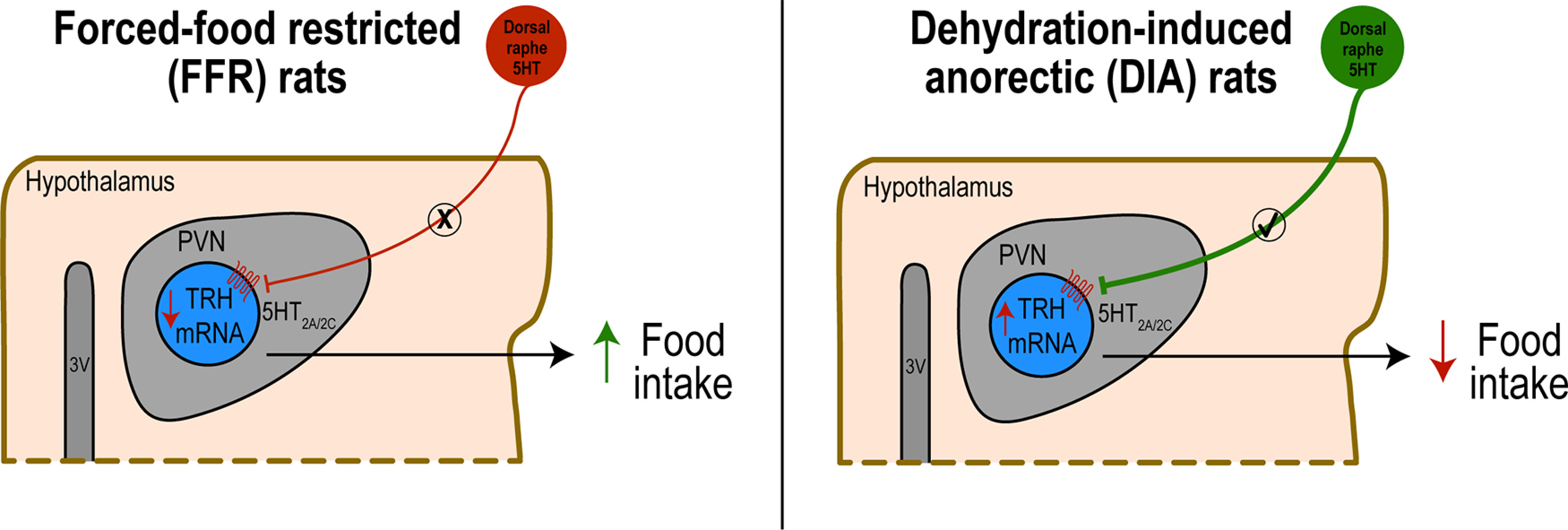

## Significance Statement

Interaction between brain peptides and neurotransmitters’ pathways regulates feeding behavior, but when altered it could lead to the development of eating disorders, such as anorexia. An abnormal increased thyrotropin-releasing hormone (TRH) expression in hypothalamic paraventricular nucleus (PVN) results in dehydration-induced anorectic rats, associated to their low food intake. The role of neurotransmitters in that alteration is unknown, and since serotonin inhibits feeding and has receptors in PVN, we analyzed its participation in increasing TRH expression and reducing feeding in anorectic rats. By antagonizing PVN serotonin receptors in anorectic rats, we identify decreased TRH expression and increased feeding, suggesting that the anorectic effects of serotonin are mediated by PVN TRH. Elucidating brain networks involved in feeding regulation would help to design therapies for eating disorders.

## Introduction

Anorexia nervosa is a psychiatric eating disorder characterized by decreased food intake even when individuals present a loss of >30% of their ideal weight. The disorder involves development of malnutrition with muscle mass loss (Casper et al., 2020) because of a negative energy balance (NEB), which seems unable to activate patients’ appetite.

Given that different peripheral and central signals are involved in activating feeding in conditions of NEB, there are many potential candidate molecules as responsible for the impaired appetite stimulation that anorectic individuals exhibit. Efforts have been made to elucidate whether information from peripheral hormones (leptin or insulin) and the response of hypothalamic factors to NEB, are defective and unable to signal to the brain of anorectic patients about their depleted energy stores and to activate feeding centers (Tortorella et al., 2014).

To analyze some of the impaired responses of the hypothalamus to the extenuated energy stores in anorexia, animal models that mimic the decreased food intake of patients have been used. Among those, there is the dehydration-induced anorexia (DIA) model that consists in exposing rats to a 2.5% NaCl solution as a unique liquid source for 5 d, which progressively inhibits food intake as a behavioral adaptation to dehydration (Watts, 1998). Anorectic behavior is displayed despite the dramatic weight loss that rats show and of the activation of arcuate (ARC) orexigenic signals. This adaptation privileges the maintenance of intracellular and extracellular water levels avoiding its use for digestion, and limits osmolytes intake from food (Watts et al., 1999). Besides dehydrated rats (DIA), the model includes another group of animals that are forced to reduce their food consumption [forced-food restricted (FFR)] to the amount eaten by DIA; in consequence, FFR rats are hungry and would increase their food intake if it was offered.

The use of this model has helped to unravel the organization of the networks that regulate feeding behavior, and it has been identified that second-order neurons of the hypothalamic paraventricular nucleus (PVN) that synthesize thyrotropin-releasing hormone (TRH), implicated with anorectic effects (Vijayan and McCann, 1977; Suzuki et al., 1982; Choi et al., 2002), are differentially modulated in DIA and FFR animals. FFR rats show decreased expression of TRH in the PVN consistent with their hunger and NEB (Blake et al., 1991; Van Haasteren et al., 1995), whereas DIA animals exhibit an increase in TRH mRNA content, suggesting a role for TRH in their anorectic behavior (Jaimes-Hoy et al., 2008; de Gortari et al., 2009; García-Luna et al., 2010; Alvarez-Salas et al., 2012).

PVN TRHergic neurons receive different innervation of neuropeptides and neurotransmitters that may be involved in the increased PVN TRH expression of dehydrated animals and in maintaining its anorectic behavior, even when peripheral signals and ARC peptides respond adequately to their low lipid stores.

A neurotransmitter that is possibly involved in the aberrant increase of TRH expression is serotonin (5-hydroxytryptamine; 5HT), since it has anorectic effects that are well described (Fletcher and Burton, 1986; Leibowitz, 1990; Stallone and Levitsky, 1994; Vickers et al., 2001). For example, 5HT peripheral administration reduces rats’ feeding and the blockade of its effects with antagonists directly injected into the PVN, identifies this hypothalamic nucleus as the key site for controlling its anorectic actions (Weiss et al., 1986; Leibowitz and Alexander, 1991). Importantly, there is a high expression of 5HT_1_, 5HT_2_, 5HT_6_ receptors in the PVN (Marvin et al., 2010; Garfield et al., 2014; Lauffer et al., 2016), and the anorectic effects of the serotonergic pathway result by activating 5HT_1_ and 5HT_2_ receptors (Bagdy, 1996; Doslikova et al., 2013), or by the antagonism of 5HT_6_ (Woolley et al., 2001). Furthermore, food deprivation-induced hungry animals show reduced 5HT concentration in their brain (Haleem, 2009), whereas those with anorexia present high c-fos expression in different areas including the PVN that is blocked with 5HT antagonists (LPS-induced anorectic rats; Kopf et al., 2010), a serotonergic hyperinnervation (anx/anx mice; Jahng et al., 1998), or an enhanced 5HT turnover (stress-induced anorectic rats; Reed et al., 2021) in the hypothalamus.

The above information coupled with data showing that serotonin can increase TRH expression in cardiomyocytes (Shi et al., 1997) and its release from median eminence *in vitro* (Chen and Ramirez, 1981), led us to explore a possible role of 5HT in inducing the anorectic behavior of dehydrated rats by increasing TRH mRNA levels in their PVN. Therefore, we initially investigated the expression of 5HT receptors known to be present in the PVN and involved in serotonin-inhibiting feeding actions using the adult mouse hypothalamic cell line mHypoA-2/30. In parallel, we studied the intracellular pathways involved in increasing TRH mRNA by 5HT in this cell line. Then, we studied the 5HT role in regulating TRH mRNA content in the PVN of DIA rats and compared it to control and FFR animals. To achieve that, we targeted 5HT_2A/2C_ receptors activity by injecting specific antagonists or agonists into the PVN of DIA or FFR rats, respectively, and analyzed a possible attenuation of the anorexia in DIA or a reduced appetite in re-fed FFR animals and its association with changes in PVN TRH mRNA levels.

## Materials and Methods

### *In vitro* studies

#### Cultured hypothalamic cells

Adult mouse hypothalamic neuronal cell line mHypoA-2/30 (CELLutions Biosystems Inc) was cultured to analyze the effect of 5HT on TRH expression and signaling pathways of 5HT_1A_, 5HT_2C_, and 5HT_6_ receptors. Cells were grown in six-well plates (Corning Life Sciences) with high glucose DMEM (4500 mg/l; Sigma-Aldrich) supplemented with 5% fetal bovine serum (FBS) and 1% penicillin-streptomycin (Biochrome AG) and maintained at 37°C and 5% CO_2_ until 90% confluency.

Immortalized mHypoA-2/30 cells were treated with vehicle (water), 10 or 100 μm of 5HT (Sigma-Aldrich) for 24 h. In another experiment, cells were treated with the PKA inhibitor H89 (PKAi, 10 μm, Tocris Bioscience) or the PKC inhibitor chelerythrine chloride (PKCi, 1 μm, Sigma-Aldrich) 15 min before addition of 100 μm of 5HT and incubated for 24 h. Medium was then aspired and the GenElute mammalian total RNA miniprep kit (Sigma-Aldrich) used to extract total RNA from the samples.

#### Immunoblotting

In another set of experiments, the hypothalamic cell line was treated with 10 μm of PKAi or 1 μm of PKCi for 1 h and then treated with vehicle or 100 μm of 5HT for 5 min. After medium removal and a PBS wash, RIPA lysis buffer (Abcam) with protease and phosphatase inhibitors (1:100, Thermo Fisher Scientific) was used to extract proteins. Cell lysates were collected and centrifuged at 10,400 × *g* for 15 min. Supernatants were collected and a 10 μl aliquot was used for protein determination by the Pierce BSA protein assay kit (Thermo Fisher Scientific). The rest of the sample was diluted with the same volume of 2× Laemmli buffer (Sigma-Aldrich) and denatured at 95°C for 5 min.

Protein samples (20 μg) were loaded in 10% SDS-PAGE and after 1 h, transferred to nitrocellulose membranes (GE Healthcare). Membranes were then incubated with blocking solution (GE Healthcare) during 1 h and incubated overnight with the primary antibodies for phospho-CREB (pCREB; Ser 133, 1:750; 9198 Cell Signaling Technology), total CREB (1:1000; 4820 Cell Signaling Technology), phospho-ERK 1/2 (pERK1/2; Thr202/Tyr204, 1:1000; 4377 Cell Signaling Technology), total ERK 1/2 (1:1000; 4696 Cell Signaling Technology) in 3% BSA. After washings with Tris-buffered saline 0.1% Tween, membranes were incubated for 1 h with the secondary antibody (1:20,000 anti-rabbit DyLight 800 conjugate; 5151 Cell Signaling Technology or 1:30,000 anti-mouse DyLight 680 conjugate; 5470 Cell Signaling Technology) in 3% BSA. Protein bands were then visualized with the Odyssey infrared imaging system (LICOR Biosciences). ImageJ software (National Institutes of Health) was used for the analysis and quantification of fluorescent signals.

### *In vivo* studies

#### Animals

Female Wistar rats weighing 230–260 g were obtained from the Psychiatric National Institute’s (INPRFM) animal house and experiments were approved by the INPRFM’s Projects Commission and Ethics Committee following the regulations established by the Mexican Official Norm for the use and care of laboratory animals NOM-062-ZOO-1999. Animals were housed in individual cages under standard environmental conditions (lights on from 6:30 A.M. to 6:30 P.M.), temperature 24 ± 1°C, maintained with *ad libitum* access to food (Rodent Laboratory Chow 5001, PMI Nutrition International) and tap water for one week to allow their adaptation to the housing conditions. All rats were cannulated into the PVN by stereotaxic surgery and allowed to recover for 5 d until they started to consume the amount of food eaten before surgery.

On a first trial, rats were randomly assigned to three control groups: C-veh, C-DOI and C-KTN which were injected with saline solution (veh), with 5HT_2A/2C_ agonist 2,5-dimethoxy-4-iodoamphetamine (DOI) or with the 5HT_2_ antagonist ketanserin (KTN) into the PVN (*n* = 10/group). On a second trial, cannulated rats were subjected to the DIA paradigm (described below) and on the last day of the experiment, a third of the control animals were injected with vehicle (C-veh), with DOI (C-DOI), or with KTN (C-KTN), half of the animals of DIA group were injected with vehicle (DIA-veh) or with KTN (DIA-KTN, *n* = 7–10/group) and FFR were injected with vehicle (FFR-veh) or with DOI (FFR-DOI, *n* = 6–8/group) and then divided into two subgroups. The first subgroup with no refeeding period included C, DIA and FFR animals injected on the fifth day with vehicle or drugs (*n* = 4–6/group) at 6:30 P.M. and euthanized at 7:00 P.M.; the second subgroup with re-feeding period included C, DIA and FFR animals (*n* = 4–6/group) injected as the first subgroup but at 7:00 P.M. they were offered with food and allowed to eat for 1 h; quantity of food eaten was recorded and then all animals were killed immediately. Brains were excised and immediately frozen at −70°C for analyzing TRH mRNA expression in their hypothalamic PVN.

#### Surgery

Rats were anesthetized with 100 mg/kg of ketamine (Anesket, PISA Agropecuaria) and 10 mg/kg of intramuscular xylazine (Procin, PISA Agropecuaria). After being placed in a stereotaxic apparatus, rats were unilaterally implanted with a stainless-steel guided cannula (20-gauge, BD Medical), secured 3.5 mm above the PVN [anteroposterior (AP): −1.6; lateral (L): +0.3 from bregma; dorsal ventral (DV): −4 from the skull; Paxinos and Watson, 2005]; reaching the DV: −7.5 mm with the injector. After 5 d of recovery and normalization of their food intake, animals were treated as described above. The injection site was verified with a scanned image (Scanner HP 5550) of a 60-μm medial PVN slice (−1.44 mm from bregma) of a rat injected with blue ink (Extended Data Fig. 3-1).

**Figure 1.**
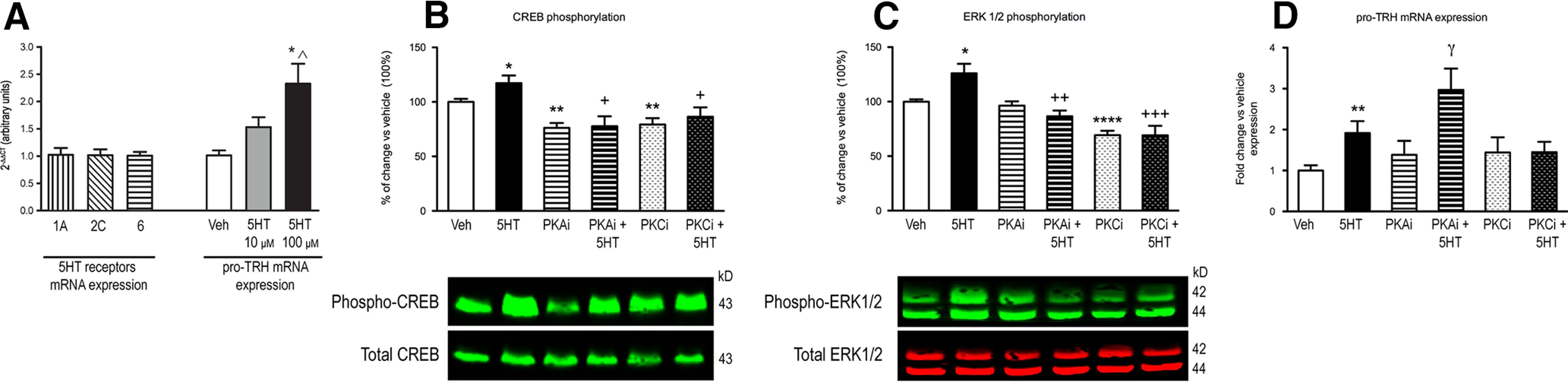
Expression of 5HT receptors and effects of 5HT, PKA, and PKC inhibitors on intracellular second messengers’ content and in pro-TRH mRNA levels in mHypoA-2/30 cells. ***A***, 5HT_1A_, 5HT_2C_, and 5HT_6_ receptors basal mRNA expression and pro-TRH mRNA levels in cell cultures treated with vehicle or different concentrations of 5HT for 24 h. Data represent the mean ± SEM of the mRNA levels expressed in arbitrary units (*n* = 3 wells/treatment). One-way ANOVA of pro-TRH mRNA expression showed a significant effect of treatment: *F*_(2,9)_ = 7.478, *p < 0.05 versus veh, ^^^*p* < 0.01 versus 5HT 10 μm; (***B***) pCREB and (***C***) pERK1/2 content in cell cultures treated with vehicle or 5HT (100 μm) during 5 min, with the PKA inhibitor H89 (PKAi, 10 μm) or the PKC inhibitor chelerythrine chloride (PKCi, 1 μm) for 1 h, or with the PKAi or PKCi for 1 h and then with 5HT for 5 min (*n* = 4–5 wells/treatment). Values are expressed as percent of change versus vehicle-treated cultures’ values (100%). For pCREB content, the two-way ANOVA showed a significant effect of 5HT: *F*_(1,40)_ = 5.25, *p* < 0.05, or of treatment with inhibitors: *F*_(2,40)_ = 13.65, *p* < 0.001; *p < 0.05, **p < 0.01 versus veh, ^+^*p* < 0.05 versus 5HT. For pERK1/2, the two-way ANOVA showed a significant effect of 5HT: *F*_(1,38)_ = 4.45, *p* < 0.05, or of treatment with inhibitors: *F*_(2,38)_ = 27.25, *p* < 0.0001 and an interaction between inhibitors and serotonin treatments: *F*_(2,38)_ = 8.44, *p* < 0.0001; *p < 0.05, ****p < 0.0001 versus veh, ^++^*p* < 0.01, ^+++^*p* < 0.001 versus 5HT. ***D***, pro-TRH mRNA expression in cell cultures treated with vehicle, 5HT (100 μm), PKAi (10 μm), PKCi (1 μm), or PKAi or PKCi and 5HT for 24 h (*n* = 5–10 wells/treatment). Data are expressed as the mean ± SEM of the fold change compared with vehicle values (1). The two-way ANOVA showed a significant effect of 5HT: *F*_(1,43)_ = 10.27, *p* < 0.01, or inhibitors treatment: *F*_(2,43)_ = 3.30, *p* < 0.05; **p < 0.01 versus veh; ^ɣ^*p* < 0.05 versus PKAi. PKA: protein kinase A, PKC: protein kinase C, pCREB: phosphorylated cAMP response element binding protein, pERK: phosphorylated extracellular signal regulated kinase, SEM: standard error of the mean, veh: vehicle, 5HT: serotonin.

10.1523/ENEURO.0077-22.2022.f3-1 Extended Data Figure 3-1 Scanned image of a representative brain slice showing the injection site at medial PVN. Digital picture of a medial fresh slice (Scanner HP 5550; −1.44 mm from bregma) showing blue ink in the PVN. Download Figure 3-1, TIF file.

#### DIA model

We followed the DIA model described by Watts et al. (1999). All cannulated and recovered rats were randomly assigned to one of the 3 following groups (*n* = 10/group) and subjected to the experimental conditions for 5 d: (1) control (C) group, which was offered food and water *ad* *libitum* for 5 d; (2) dehydration-induced anorectic animals (DIA), which were allowed to consume as drinking liquid only a 2.5% NaCl solution and had *ad libitum* access to food; (3) FFR group receiving tap water *ad libitum* and a similar amount (the average of DIA’s food intake for two previous trials) of food ingested by DIA rats (Jaimes-Hoy et al., 2008). Food intake and body weight of all groups were measured daily up to the morning of the fifth day before being injected. As described above, on the fifth day at 6:30 P.M., animals were injected with vehicle, DOI, or KTN; then, half of each group was killed at 7:00 P.M. with no re-feeding, whereas the other half was re-fed at 7:00 P.M. for 1 h and decapitated immediately after.

### qRT-PCR

Frozen PVN (0.84 mm to −2.04 mm from bregma) were punch-dissected from coronal slices using a 1 mm diameter sample corer and homogenized in 4 m guanidine thiocyanate (ICN). Total RNA was extracted as described elsewhere (Chomczynski and Sacchi, 1987). RNA quality of samples was verified by the O.D. absorbances of 260/280 and 260/230 nm ratios and by quantifying 28S/18S relation using agarose gel electrophoresis; samples were discarded when the ratio was not higher than 1.5; 1.5 μg of RNA and oligo dT (100 pmol/ml; Biotecnologías Universitarias, Universidad Nacional Autónoma de México, Cuernavaca, Morelos, México) were used to obtain cDNA (M-MLV reverse transcriptase, Invitrogen). We quantified pro-TRH mRNA levels by RT-PCR using TaqMan Universal PCR Master Mix (Applied Biosystems) on a 7500 real-time PCR system (Thermo-Fisher) and oligonucleotide primers: *Trh* (Rn00564880_m1, Applied Biosystems). Each PCR was performed in duplicate; pro-TRH mRNA expression levels were normalized using β-actin (*Actb*; Rn00667869_m1) as housekeeping control gene.

For the determination of genes expression in the hypothalamic cell line, cDNA was synthetized using the High-Capacity RNA to cDNA kit (Applied Biosystems) for 1 h at 37°C. PCRs were performed using the Sensi FAST SYBR Lo-ROX kit reagents (Bioline) and oligonucleotide primers: *Trh* (forward: 5′-GCTCTGGCTTTGATCTTCGTG-3′, reverse: 5′-CCGGACCTGGACTTTCTCC-3′), *5HT_1A_* (forward: 5′-GACAGGCGGCAACGATACT-3′, reverse: 5′-CCAAGGAGCCGATGAGATAGTT-3′), *5HT_2C_* (forward: 5′-GATGGTGGACGCTTGTTTCAA-3′, reverse: 5′-GCCATGATAACGAGAATGTTGC-3′), *5HT_6_* (forward: 5′-GCTGTGCGTGGTCATCGTA-3′, reverse: 5′-CATCAGGTCCGACGTGAAGAG-3′), and *Actb* (forward: 5′-GGCTGTATTCCCCTCCATCG-3′, reverse: 5′-CCAGTTGGTAACAATGCCATGT-3′).

Changes of different mRNAs content in the PVN tissue and in the cell line were evaluated from the threshold cycle using the ΔCt method; the ΔCt value was obtained from the difference between the number of cycles for each problem gene and that of *Actb* to reach the threshold (ΔCt = Ct of unknown genes− Ct *Actb*). Groups used as reference were the control injected with vehicle or vehicle-treated cells, whose value of fluorescence in arbitrary units was considered as 1 and the percentage of change for each gene examined was obtained by the equation:

$$\Delta \Delta \text{Ct }={\text{ 2}}^{-\left(\Delta \text{Ct}=\text{Ct Control }–\text{Ct experimental group}\right)}$$

### Statistics

Changes in body weight and food intake of animals before receiving injections of the described drugs and subjected to the DIA paradigm were analyzed by one-way repeated measures ANOVA. PVN TRH mRNA expression and food intake of vehicle-injected or drugs-injected animals from different groups were analyzed by two-way ANOVA (treatments × groups); comparisons of genes and proteins expression between treatments of hypothalamic cultured cells were analyzed by one-way (different 5HT doses) and two-way (5HT × inhibitors treatment) ANOVA, respectively, using the Stat-View software (SAS Institute). When *p* was <0.05 a Tukey’s *post hoc* test was performed.

## Results

### *In vitro* studies in mHypoA-2/30 cells

In preliminary experiments, we identified the expression of 5HT_1A_, 5HT_2C_, and 5HT_6_ in mHypoA-2/30 cells (Fig. 1*A*) and observed that addition of 10 or 100 μm of 5HT increased the mRNA levels of pro-TRH by 53% and 130%, respectively, compared with control cultures (Fig. 1*A*).

As 5HT_1A_, 5HT_2C_, 5HT_6_ transduce signals through PKA or PKC-induced phosphorylation of CREB or ERK and as TRH gene promoter has consensus sites for pCREB and pERK1/2, we analyzed the effect of adding 5HT on pCREB or pERK levels in the presence or absence of PKA or PKC inhibitors (PKAi, PKCi). 5HT induced a modest but statistically significant stimulation of pCREB by 17% when compared with control, which was abolished in the presence of PKAi or PKCi (Fig. 1*B*).

5HT also increased pERK1/2 levels by 26% above basal; addition of PKCi alone or in combination with 5HT blocked 5HT-induced increase of pERK levels. Addition of PKAi exerted similar effects on 5HT-induced pERK1/2 (Fig. 1*C*). These results suggested that 5HT-induced CREB and ERK1/2 phosphorylation required intact PKA and PKC activities.

Figure 1*D* shows the increase in pro-TRH mRNA levels by 92% above basal after treatment with 100 μm of 5HT. Basal TRH mRNA expression was not affected by adding PKAi or PKCi. However, PKCi blocked the effect of 5HT on TRH mRNA levels; in contrast, mHypoA-2/30 cells treated with PKAi showed an enhanced response to 5HT, showing an increase in TRH mRNA levels by 190%, more than twice compared with 5HT response in the absence of any kinase inhibitor.

### *In vivo* studies: DIA model

**Figure 2.**
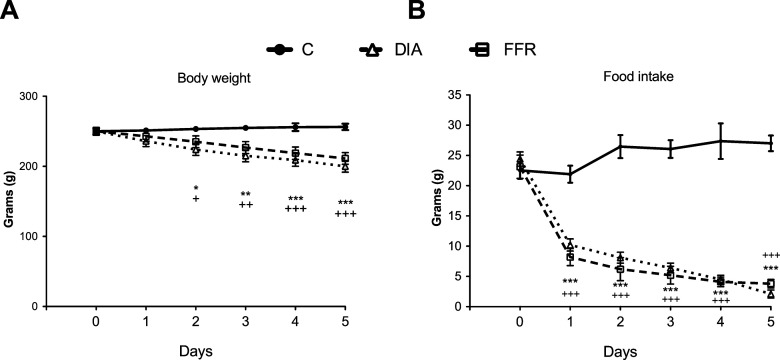
Daily body weight and food intake registers. Body weight (***A***) and food consumption (***B***) in C, DIA and FFR rats from 0 to 5d. Data are expressed as the mean ± SEM in grams, *n* = 10 per group. For body weight the one-way repeated measures ANOVA showed differences through time *F*_(5,162)_ = 5.436, *p* < 0.001, between groups: *F*_(2,162)_ = 126.434, *p* < 0.001 and a significant interaction between time and groups: *F*_(10,162)_ = 3.139, *p* < 0.001. For food intake, the one-way repeated measures ANOVA showed a significant effect of time: *F*_(2,162)_ = 66.219, *p* < 0.001; of the interaction between time and groups: *F*_(10,162)_ = 2.091, *p* < 0.05). *p < 0.05, **p < 0.01, ***p < 0.001 DIA versus C rats. ^+^*p* < 0.05, ^++^*p* < 0.01, ^+++^*p* < 0.001 FFR versus C rats. SEM: standard error of the mean, C: control, DIA: dehydration-induced anorexia, FFR: forced-food restriction.

On the second day of subjecting rats to the dehydration model, DIA group lost 12% of body weight and on the fifth day 22% when compared with C group (100%, second day: 253 ± 2.6 g; fifth day: 256 ± 4.5 g). FFR rats also decreased their body weight, when compared with the C group, similarly to DIA animals (Fig. 2*A*).

DIA group reduced their food intake by 53% compared with C after 24 h of dehydration, and by the fifth day they decrease 92% of the amount ingested by C (100%, first day: 22 ± 1.4 g; fifth day: 27 ± 1.3 g; Fig. 2*B*).

### Effect of DOI and KTN on food intake and PVN TRH expression of control animals

**Figure 3.**
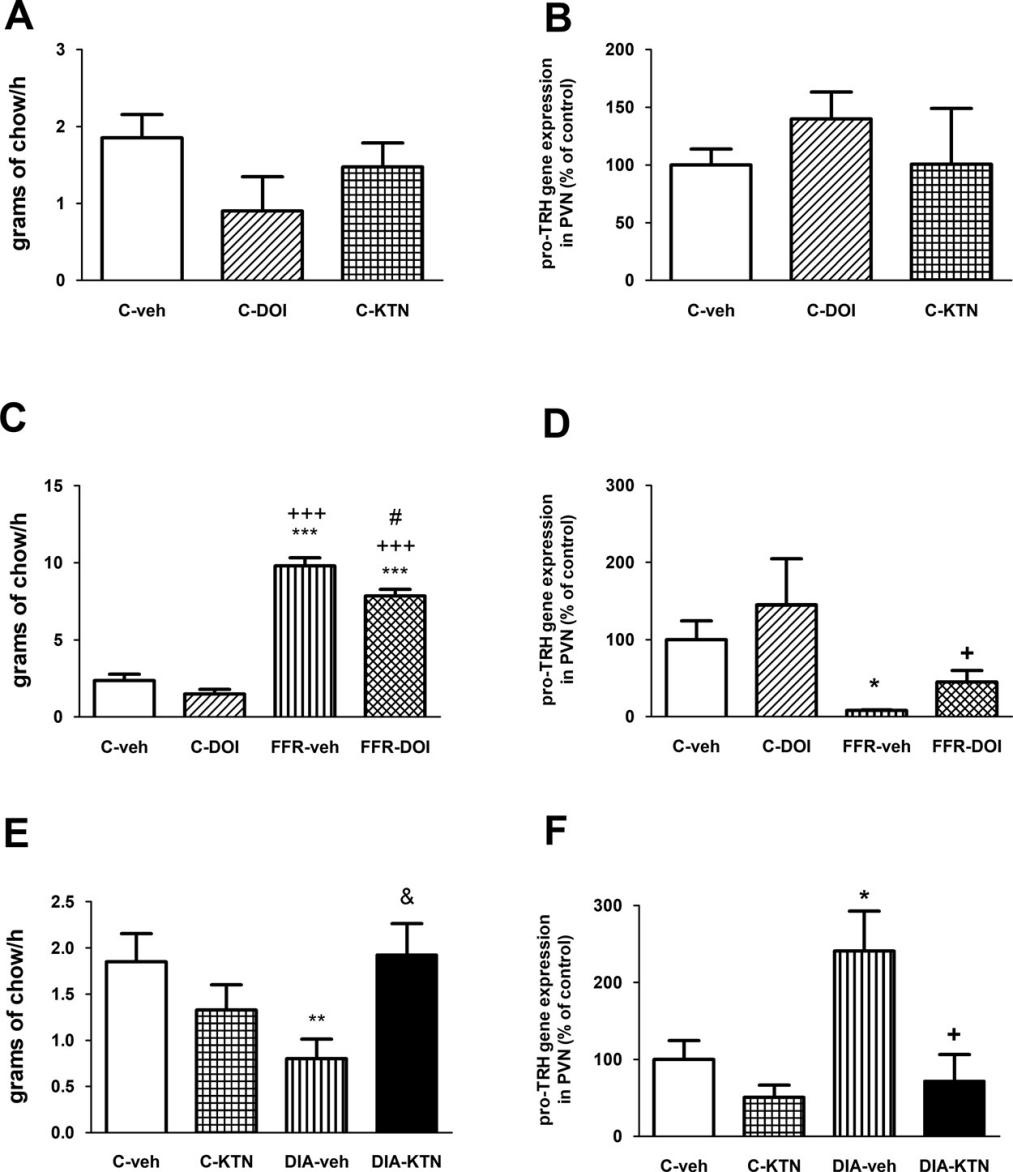
Effect of the administration of DOI or KTN to female rats on their food intake and PVN TRH mRNA levels. ***A***, Food consumption of C rats injected with veh, DOI, or KTN (Extended Data Fig. 3-1) and after a re-fed period of 1 h. ***B***, PVN pro-TRH mRNA expression of C rats after 30 min of the injection with veh, DOI, or KTN. ***C***, Food consumption of C and FFR rats injected with veh or DOI and after a re-fed period of 1 h. ***D***, PVN pro-TRH mRNA expression of C and FFR rats after 30 min of the injection with veh or DOI. ***E***, Food consumption of C and DIA rats injected with veh or KTN and after a re-fed period of 1 h. ***F***, PVN pro-TRH mRNA expression of C and DIA rats after 30 min of the injection with veh or KTN. PVN TRH mRNA levels are plotted as percent of change versus vehicle animals (100%). Values indicate the mean ± SEM. ***C***, Two-way ANOVA showed significant effect of groups: *F*_(1,14)_ = 233.44, *p* < 0.001, and treatment: *F*_(1,14)_ = 9.93, *p* < 0.01. ***D***, Two-way ANOVA showed a significant interaction between groups and treatment: *F*_(1,25)_ = 6.78, *p* < 0.05. ***E***, Two-way ANOVA showed differences between groups: *F*_(1,12)_ = 8.38, *p* < 0.05. ***F***, Two-way ANOVA showed differences between treatments: *F*_(1,13)_ = 8.34, *p* < 0.05. ^#^*p* < 0.05 FFR-DOI versus FFR-veh; ^&^*p* < 0.05 DIA-KTN versus DIA-veh, *p < 0.05 FFR-veh or DIA-veh versus C-veh rats, ^+^*p* < 0.05 FFR-DOI or DIA-KTN versus FFR-veh or DIA-veh rats, respectively, **p < 0.01 DIA versus C-veh rats, ***p < 0.001 FFR-veh or FFR-DOI versus C-veh rats, ^+++^FFR or FFR-DOI versus DOI animals. SEM: standard error of the mean, C: control, DIA: dehydration-induced anorexia, FFR: forced-food restriction, veh: vehicle, DOI: 5HT agonist, KTN: 5HT antagonist.

We investigated whether the 5HT agonist (DOI) and antagonist (KTN) were able to induce changes in PVN TRH mRNA expression and food intake in C rats after 30 min of being injected. We observed no significant changes in food intake (Fig. 3*A*) or in PVN TRH mRNA levels (Fig. 3*B*) between C-veh, C-DOI, and C-KTN groups.

### Food intake of FFR and DIA groups after injecting DOI or KTN, respectively

FFR animals were offered a very low amount of food and became hungry since they were pair-fed with anorectic rats that reduce their appetite; thus, on the afternoon of day 5, when food was offered *ad libitum* for 1 h, FFR-veh consumed 387% more food than C-veh (100%, 2.4 ± 0.4 g; Fig. 3*C*). Also, the FFR-DOI group increased the amount of food eaten to 337%, when compared with the C-veh group but showed a reduction of 13% when compared with FFR-veh group (Fig. 3*C*). Figure 3*E* shows that DIA-veh rats reduced their food intake 33% compared with C-veh. In contrast, the anorectic behavior of the DIA-KTN group was reversed and consumed a similar amount of food compared with C-veh (100%, 2.4 ± 0.4 g).

### TRH expression in the hypothalamic PVN of FFR and DIA animals after DOI or KTN injection, respectively

In order to analyze the effect of DOI and KTN injection on PVN TRH mRNA expression of DIA and FFR groups of rats, respectively, on the day 5 rats with no re-fed period were killed 30 min after injection with vehicle or drugs. Figure 3*D* shows that the injection of DOI to controls (C-DOI) did not change TRH expression when compared with controls injected with vehicle (C-veh), but TRH mRNA in the PVN showed a 92% decrease in FFR-veh compared with the C-veh group. DOI administration to FFR group (FFR-DOI) partly reversed this decrease in TRH expression observed in FFR-veh, showing a fivefold increase compared with FFR-veh group.

Figure 3*F* shows that PVN TRH mRNA expression increased in DIA-veh by 241% compared with C-veh, and that the injection of KTN in DIA group abolished the DIA effect and in fact reduced TRH mRNA expression to similar levels of those of C-veh.

## Discussion

Anorectic effects of TRH are well documented; when subcutaneously, intravenously, or intracerebroventricularly injected, rodents inhibit their food intake (Vijayan and McCann, 1977; Suzuki et al., 1982; Choi et al., 2002); however, the more studied function of the PVN TRH is its role in the regulation of the hypothalamic-pituitary-thyroid (HPT) axis. This axis controls energy homeostasis and expenditure; for example, fasting, malnutrition, or weight loss induce an axis adaptation leading to a reduced TRH expression in the PVN, which decelerates the HPT axis function and declines the fuel degradation rate, allowing animals to cope with their low nutrient availability (Blake et al., 1991; Van Haasteren et al., 1995).

As a compensatory mechanism to the NEB, appetite of animals increases because of the activation of ARC orexigenic signals (NPY/AgRP) and the inhibition of the anorectic ones (POMC/CART). PVN TRHergic neurons receive these feeding regulatory afferent projections from the ARC (Légrádi and Lechan, 1998, 1999; Fekete et al., 2000a,b), which in turn induce a decreased TRH expression favoring the food seeking behavior of rats. The reduced TRH mRNA levels in the PVN of fasting-induced hungry animals support its role as an anorectic peptide.

Interestingly, DIA rats do not present the expected decrease in TRH expression in the PVN despite their NEB, implicating it in the development of their anorectic behavior (Jaimes-Hoy et al., 2008; Alvarez-Salas et al., 2012).

PVN TRHergic neurons not only receive afferent signals from the ARC, but also from feeding-regulatory pathways of different brain regions. Thus, we investigated here the role of serotonin pathway in promoting PVN TRH expression of dehydrated rats, favoring their anorectic behavior. This is supported by 5HT-induced increase in TRH expression in *in vitro* systems (Chen and Ramirez, 1981; Shi et al., 1997) and by the high expression of 5HT receptors in the PVN (Marvin et al., 2010; Garfield et al., 2014; Lauffer et al., 2016) that when activated by 5HT injection, induce a reduction of food intake in rodents (Weiss et al., 1986; Leibowitz and Alexander, 1991; Bagdy, 1996; Woolley et al., 2001; Doslikova et al., 2013).

### *In vitro* study

Studies on G-protein-coupled receptors pathways of 5HT receptor subtypes have demonstrated that 5HT_1A_ is coupled to G_i_ and 5HT_6_ to G_s_, thus, their stimulation modulates CREB phosphorylation through the regulation of PKA activity (Raymond et al., 1999; Albert and Robillard, 2002). In turn, 5HT_2A/2C_ receptors are coupled to G_q_ and downstream they can activate the PKC-ERK1/2 intracellular pathway (Albert and Vahid-Ansari, 2019). Interestingly, TRH promoter gene has consensus sites for both pCREB and for PKC-induced AP1 transcription factor (Lee et al., 1988).

As expected, we observed an increase in pERK and pCREB levels when 5HT was added to cultures given that 5HT_2C_ and 5HT_6_ are expressed in the cell line used. Since the blockade of PKA or PKC prevented 5HT effect on the second messengers pERK and pCREB, our results are in agreement with *in vivo* and *in vitro* studies, showing that CREB and ERK phosphorylation is regulated by the crosstalk between PKA and PKC enzymes activities (Roberson et al., 1999; Mao et al., 2007). In fact, it is known that PKA activation by 5HT_6_ receptors can increase not only pCREB but also pERK levels (Hyung-Mun et al., 2007), and PKC activation by 5HT_2A_ leads to downstream phosphorylation not only of ERK1/2 but also of CREB (Chalecka-Franaszek et al., 1999). Our results suggest that 5HT signaling involves activation of PKA and PKC and downstream phosphorylation of CREB and ERK1/2.

When we evaluated the effect of those treatments on 5HT-induced TRH expression, it seemed unlikely that 5HT_1A_ was involved in TRH transcription since, as mentioned, 5HT alone was not able to decrease pCREB and TRH mRNA levels, which would be expected after activation of G_i_. Although the stimulation of 5HT_1A_ could be activating another PKC pathway, we did not find evidence of an increase in pERK1/2 levels.

The 5HT-induced pCREB expression could be because of the activation of 5HT_6_ (coupled to G_s_) since PKAi also blocked phosphorylation of CREB when induced by 5HT. However, given that 5HT was still able to increase TRH expression even in the presence of PKAi, it seemed likely that PKC transduction pathway is involved in the regulation of TRH transcription, possibly activated by additional 5HT receptor subtypes, such as 5HT_2A/2C_.

Our results support that different PKC-activated pathways other than pERK1/2 were able to regulate TRH transcription, such as AP1 that also has a consensus binding site in the TRH gene promoter (Lee et al., 1988). Furthermore, our results also support that PKA might restrain 5HT effects on increasing TRH mRNA content and might indicate that more than one 5HT type of receptors and/or signaling pathways are involved in TRH regulation. Further studies are needed to elucidate the upstream and downstream PKC-activated second messengers involved in TRH transcription.

Given that our results suggest a more likely participation of the family 2 G_q_-coupled receptors in TRH transcription, we used agonists and antagonists of 5HT_2A/2C_ receptors for the *in vivo* studies.

### *In vivo* study

#### DOI decreased food intake and increased PVN TRH mRNA levels in FFR group

The increased food intake observed in FFR-veh group when re-fed is known to result from the effects of different feeding-regulatory pathways including food restriction-induced inhibited serotonergic projections afferent to the PVN (Lam et al., 2010), lower 5HT brain concentration and the blockade of the 5HT release peak at the onset of the dark phase, which was the recording time of food intake here (Haleem, 2009). PVN discriminates the incoming signals, increasing food seeking behavior and compensating the high degradation rate of the energy stores of animals because of their NEB. Our results supported the proposed food restriction-induced attenuated 5HT afferent pathway to the PVN, since reactivation of 5HT signaling by injecting the agonist DOI to FFR in that nucleus, reduced their food intake although not to control levels.

The 5HT circadian release might explain the ineffectiveness of DOI to induce a further reduction of feeding in control rats; the partial effect of DOI in FFR supports the existence of redundant pathways that assure activation of feeding behavior in animals with NEB.

FFR-veh rats showed the expected decrease in PVN TRH mRNA levels, as it is induced by sustained NEB (Van Haasteren et al., 1995). The modest but significant increase in TRH levels of FFR-DOI group versus FFR-veh suggests that 5HT activation of its receptors in the PVN is partially needed to augment peptide’s transcription in FFR animals.

It is important to note that 5HT was able to induce TRH transcription in cell cultures, while DOI (5HT agonist) did not increase PVN TRH mRNA levels in control rats. This discrepancy could be related to the higher potency of 5HT to induce calcium release after its administration to cell cultures that express 5HT_2A_ and 5HT_2C_ receptors, than that of DOI (Seitz et al., 2012). Also, TRH expression in animals depends not only on 5HT effects but on diverse interactions with different peptides’ and neurotransmitters’ pathways (Rondeel et al., 1991; Légrádi et al., 1997; Fekete et al., 2000a,b, 2001, 2002) that are not present in cultured hypothalamic cells.

#### KTN increased feeding and decreased PVN TRH mRNA levels in the DIA group

Our findings suggest that serotonin pathway to the PVN is activated in DIA rats inducing its anorectic behavior since the blockade of PVN 5HT receptors with KTN reverses the feeding-inhibited behavior of DIA rats. Furthermore, the increased TRH expression found in DIA animals seemed facilitated by 5HT, since that change was avoided by antagonizing 5HT_2A/2C_ receptors in the PVN. Other animal models for anorexia support the involvement of 5HT_2C_ in rats’ decreased food intake: injected rats with LPS (Kopf et al., 2010), those subjected to high activity schedules (activity-based anorexia; Rieg and Aravich, 1994), or in rats subjected to a paradigm of cachexia (Ezeoke and Morley, 2015).

These results along with those found in FFR group injected with DOI confirmed a 5HT-induced inverse change between PVN TRH expression and feeding behavior, involving 5HT_2A/2C_ receptors. Other studies support 5HT_2A/2C_ receptors participation in the decreased food intake of injected rats with selective administration of 5HT agonists directly into the PVN, as well as studies in KO mice for those types of receptors (Tecott et al., 1995; Nonogaki et al., 1998; Currie et al., 2002).

As the co-expression of 5HT_2A/2C_ receptors with TRH in PVN neurons is not established, 5HT could be affecting TRH transcription directly or indirectly. Different indirect mechanisms could be involved, such as a high stimulation of 5HT_2C_ expressed in ARC-projecting terminals (Wan et al., 2020) that would inhibit NPY release into the PVN, which is known to decrease TRH transcription in this nucleus (Fekete et al., 2001). This is supported by the differential expression of NPY-Y1 in the PVN of DIA versus FFR which suggests reduced content of NPY in DIA (García-Luna et al., 2010). Another involved mechanism is a reduced expression of monocarboxylate transporters of thyroid hormones (MCT) in the mediobasal hypothalamus by 5HT, which control the T_3_ local concentration and might avoid its repression on PVN TRH transcription, as has been previously observed (Segerson et al., 1987; Seo et al., 2011; Alvarez-Salas et al., 2016). A different possibility is that a high activated serotonin pathway enhanced the signaling of lateral hypothalamic CRHergic afferents to the PVN that is specifically observed in DIA animals (Watts et al., 1999), since the blockade of CRH receptors interferes with DOI-induced feeding inhibition in control rats (Reynolds et al., 2006). That hypothesis is in agreement with studies showing that an incoming CRH is increasing PVN TRH mRNA levels, given that the blockade of CRH-R2 in the PVN attenuates TRH expression and the anorexia of DIA animals (de Gortari et al., 2009).

5HT is the first factor that reverses completely the anorectic behavior of dehydrated animals. Overall, our results supported that 5HT is favoring the decreased food intake of DIA rats and that the anorectic effects of 5HT are mediated by PVN TRH in these rats.

## Synthesis

Reviewing Editor: Alexxai Kravitz, Washington University in St. Louis Decisions are customarily a result of the Reviewing Editor and the peer reviewers coming together and discussing their recommendations until a consensus is reached. When revisions are invited, a fact-based synthesis statement explaining their decision and outlining what is needed to prepare a revision will be listed below. The following reviewer(s) agreed to reveal their identity: Ames Sutton, Paul Currie.

1) Reviewer 1 noted that the authors repeatedly mention data not shown, and make claims based on this data. This includes an important initial experiment in which expression of 5HT1A, 5HT2C and 5HT6 is demonstrated in mHypoA-2/30 cells (lines 235-236), as well as negative/control data in response to agonist and antagonist experiments in vivo (lines 268-69). Any data referenced should be shown, especially as it relates to claims made in the paper.

2) The authors use the term “food avoidance” in regards to the anorexia resulting from dehydration-induced anorexia (DIA) throughout the manuscript. Reviewer 1 noted concerns with this term that should be addressed. They suggest as an alternative that the authors can use “decreased food intake” rather than the term “food avoidance.”

3) Both reviewers noted citations that should be added to the manuscript (see full comments).

4) Reviewer 2 noted that the introduction requires a major writing, specifically to introduce the behavioral model and its importance, explain why the experiments were conducted and how they contribute to our understanding of PVN signaling and appetite. Some text could also be condensed. See full comments.

5) Several experimental details were highlighted by Reviewer 2 as lacking sufficient explanation. Please address (see full comments).

6) I am not clear if Figure 1 and Table 1 should be included in the main text as they stand. Figure 1 could be integrated into Figure 2, and the data from Table 1 into the main text or figure legends. Table 1 could be attached as a supplemental statistical results file.

Full reviewer comments:

****************

Reviewer 1

****************

Major concerns:

The authors repeatedly mention data not shown, and make claims based on this data. This includes an important initial experiment in which expression of 5HT1A, 5HT2C and 5HT6 is demonstrated in mHypoA-2/30 cells (lines 235-236), as well as negative/control data in response to agonist and antagonist experiments in vivo (lines 268-69). Any data referenced should be shown, especially as it relates to claims made in the paper. Alternatively, these statements and claims related to these experiments should be removed from the manuscript.

While the authors have performed carefully controlled experiments in the manuscript by using a FFR group to compare to DIA rats, it’s unclear why these groups aren’t all performed in agonist/antagonist experiments performed in Figure 4. To appropriately deduce how dehydration uniquely drives TRH changes via 5HT receptors, both FFR and DIA groups should be performed with KTN and DOI administration, respectively.

The authors use the term “food avoidance” in regards to the anorexia resulting from dehydration-induced anorexia (DIA) throughout the manuscript. The avoidance term suggests an aversion to food intake, rather than potentially a physiologic reaction to dehydration. While it is understandable that the authors are trying to make the correlation to anorexic individuals that actively avoid food intake, to appropriately use this term the authors should seek to prove that the rats are actively avoiding areas with food (e.g. using a conditioned place preference assay) on the DIA paradigm. Alternatively, the authors can use “decreased food intake” rather than the term “food avoidance.”

Minor concerns:

On line 70-73, the authors suggest there is evidence in the literature of 5HT and corresponding 5HT receptors in the PVN driving anorexic behavior in rats without a citation.

The authors suggest on line 73-74 that “the anorectic effects of 5HT result by activating 5HT1 and 5HT2 receptors or by the antagonism of 5HT6” in reference to Bagdy et. al 1996, and Garfield et. al 2014. These papers (in particular the Garfield et. al. 2014 paper) do not show specifically what this sentence suggests, which is that activation or antagonizing these receptors drives feeding changes. While I don’t dispute the statement made by the authors, an appropriate citation (e.g. Woolley et. al., 2001 and Bentley et. al., 1999 for 5-HT6 antagonism) would be preferred here.

It seems surprising that no changes were observed in PVN TRH mRNA in control mice (related to major concern #2 above, lines 268-69) injected with 5-HT agonists/antagonists, given the changes observed in culture in response to 5-HT presentation (Fig. 2D). This data should be shown and discussed as to the relevance and limitations of in vitro vs. in vivo results.

****************

Reviewer 2

****************

Overall, this is an interesting report investigating the role of PVN 5-HT/TRH signaling in appetite. However, the paper does require some revision.

1. The overall writing quality of the manuscript could be improved. There are numerous instances where the writing is awkward or unclear. (Example, Abstract, line 13; Intro, lines 48-51).

2. The dehydration-induced anorexia model is presented in the introduction but it is never really explained. What is this model and why is it relevant?

3. The paragraph structure of the introduction needs to be revised. The final three paragraphs could be integrated as they address the aim of the study. At the same time, it is unclear why the experiments were conducted and how they, in an integrated manner, contribute to our understanding of PVN signaling and appetite. This section needs revision.

4. It’s interesting and relevant that the experiments were conducted in female rats. Were effects observed relative to estrus? If not, is this a concern?

5. A sample size of n of 4 seems quite low. Please justify. (See top of page 7).

6. Please confirm that the ketamine dose was 100 mg/kg. This seems high for female rats. Did any rats not survive surgery?

7. A postoperative recovery of 5 days seems short. How was it determined that this time frame was sufficient?

8. Information presented in the first paragraph of the discussion would be ideally cited in the intro to provide a clearer rationale of the current report.

9. Line 386. A citation is need for the KO reference.

10. The relevance of the DIA model should, once again, be addressed in the discussion section, specifically in relation to the findings reported here.

11. Line 406: “... 5-HT is favoring the food avoiding behavior...”. What does this mean?

12. Figure 1. Cannula trajectory. I don’t see any cannula damage. Please comment.

13. What is the relevance of Table 1 and could the statistical details not be provided in the written results section alone?
