# Supplementary figures and images for: Hypothalamic TRH Mediates Anorectic Effects of Serotonin in Rats
Source: eNeuro. 2022 May 26;9(3):ENEURO.0077-22.2022. doi: 10.1523/ENEURO.0077-22.2022 (PMC9159524; doi:10.1523/ENEURO.0077-22.2022)

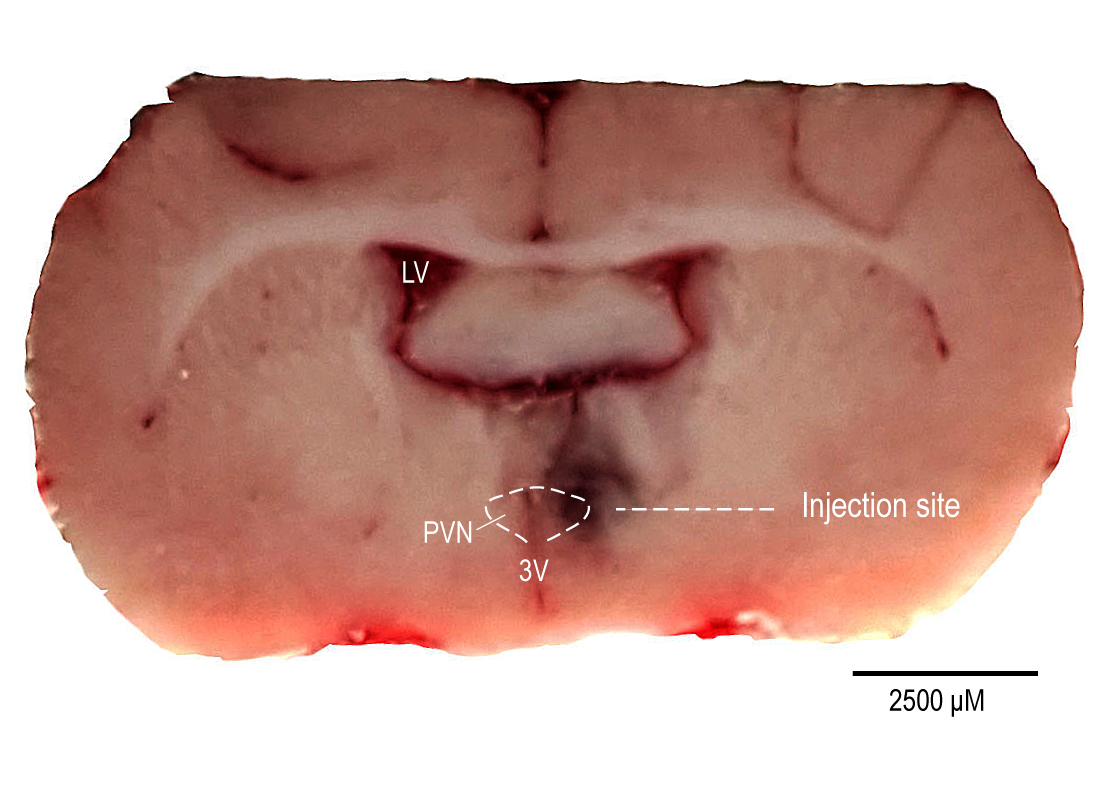

Supplement: Extended Data Figure 3-1 — Scanned image of a representative brain slice showing the injection site at medial PVN. Digital picture of a medial fresh slice (Scanner HP 5550; −1.44 mm from bregma) showing blue ink in the PVN. Download Figure 3-1, TIF file. [file enu-eN-NWR-0077-22-s03.tif]
